# Supplementary material for: Endoplasmic reticulum acetyltransferases Atase1 and Atase2 differentially regulate reticulophagy, macroautophagy and cellular acetyl-CoA metabolism
Source: Commun Biol. 2021 Apr 12;4:454. doi: 10.1038/s42003-021-01992-8 (PMC8041774; doi:10.1038/s42003-021-01992-8)
Supplement: Supplementary file 2 — Supplementary Information [file 42003_2021_1992_MOESM2_ESM.pdf]

Supplementary Information

Endoplasmic reticulum acetyltransferases Atase1 and Atase2 differentially regulate reticulophagy, macroautophagy and cellular acetyl-CoA metabolism

Michael J. Rigby, Alexis J. Lawton, Gulpreet Kaur, Varuna Banduseela, William E. Kamm, Aparna Lakkaraju, John M. Denu, and Luigi Puglielli

Contents:

- Supplemental Figures
- Supplemental Figure Legends

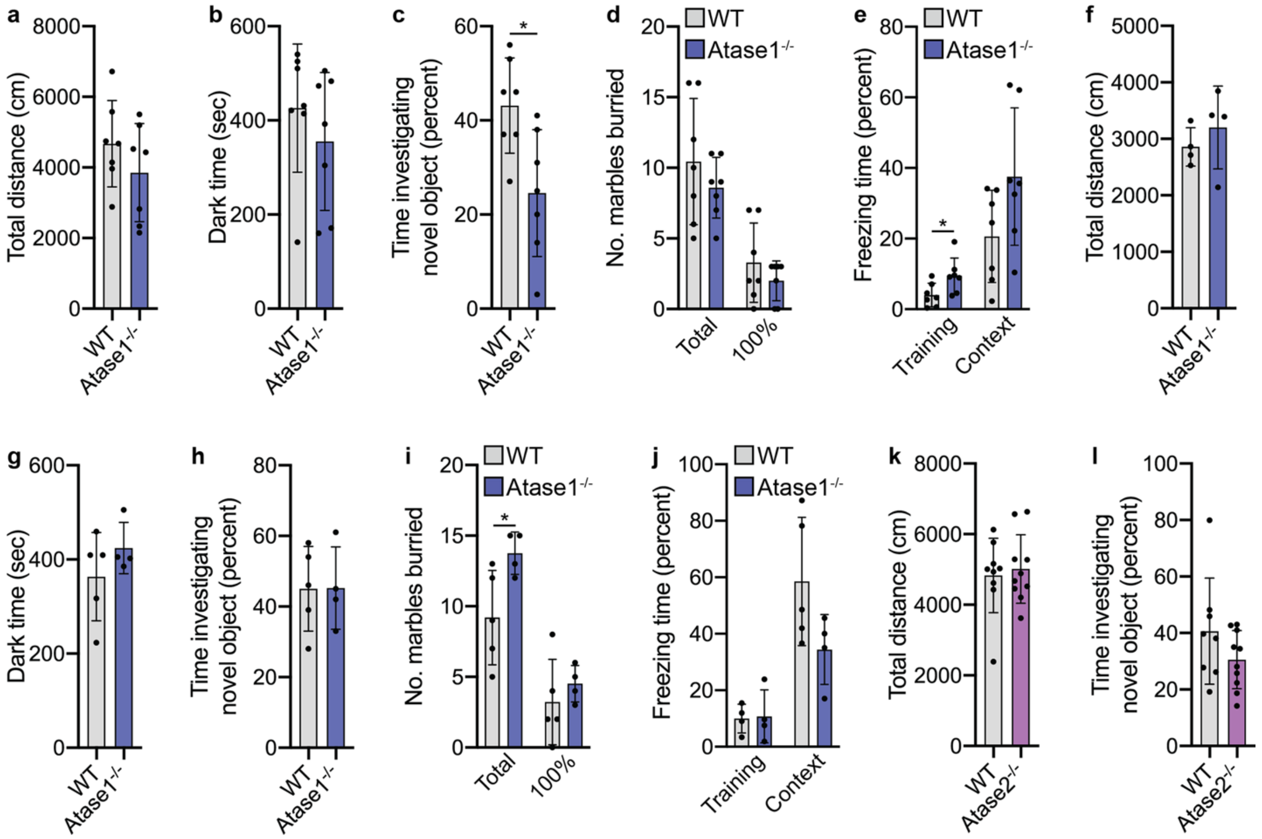

Supplemental Figure 1. Atase1$^{-/-}$ and Atase2$^{-/-}$ mice exhibit normal behavior. (a,f,k) Open field. (b,g) Light-dark box exploration. (c,h,l) Novel object recognition. (d,i) Marble burying assay. (e,j) Fear conditioning. Data are mean ± SD, *$p < 0.05$. Panels a-e are 6-month-old male mice, $n = 7$ per genotype. Panels f-j are 1-year-old female mice; WT, $n = 5$; Atase1$^{-/-}$, $n = 4$. Panels k-l are 6-month-old mice; WT, $n = 9$ (4 males); Atase2$^{-/-}$, $n = 10$ (5 males).

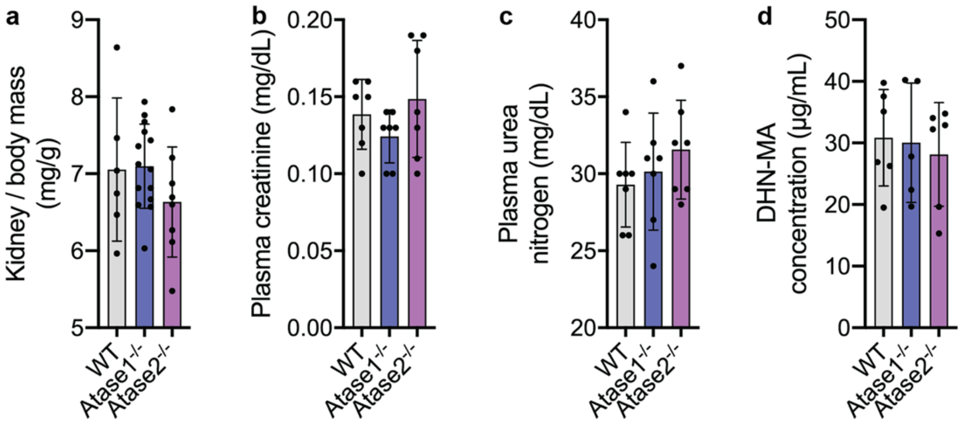

Supplemental Figure 2. Atase1$^{-/-}$ and Atase2$^{-/-}$ mice exhibit normal kidney function and mercapturic acid metabolism. (a) Kidney mass to body ratio in male WT, Atase1$^{-/-}$, and Atase2$^{-/-}$ mice at 7-9 months old. (b-c) Plasma creatinine and plasma urea nitrogen from male WT, Atase1$^{-/-}$, and Atase2$^{-/-}$ mice at 7-9 months old. (d) Spot urine 1,4-dihydroxynonane mercapturic acid (DHN-MA) from male WT, Atase1$^{-/-}$, and Atase2$^{-/-}$ mice at 3 months old. All data are displayed as mean ± SD.

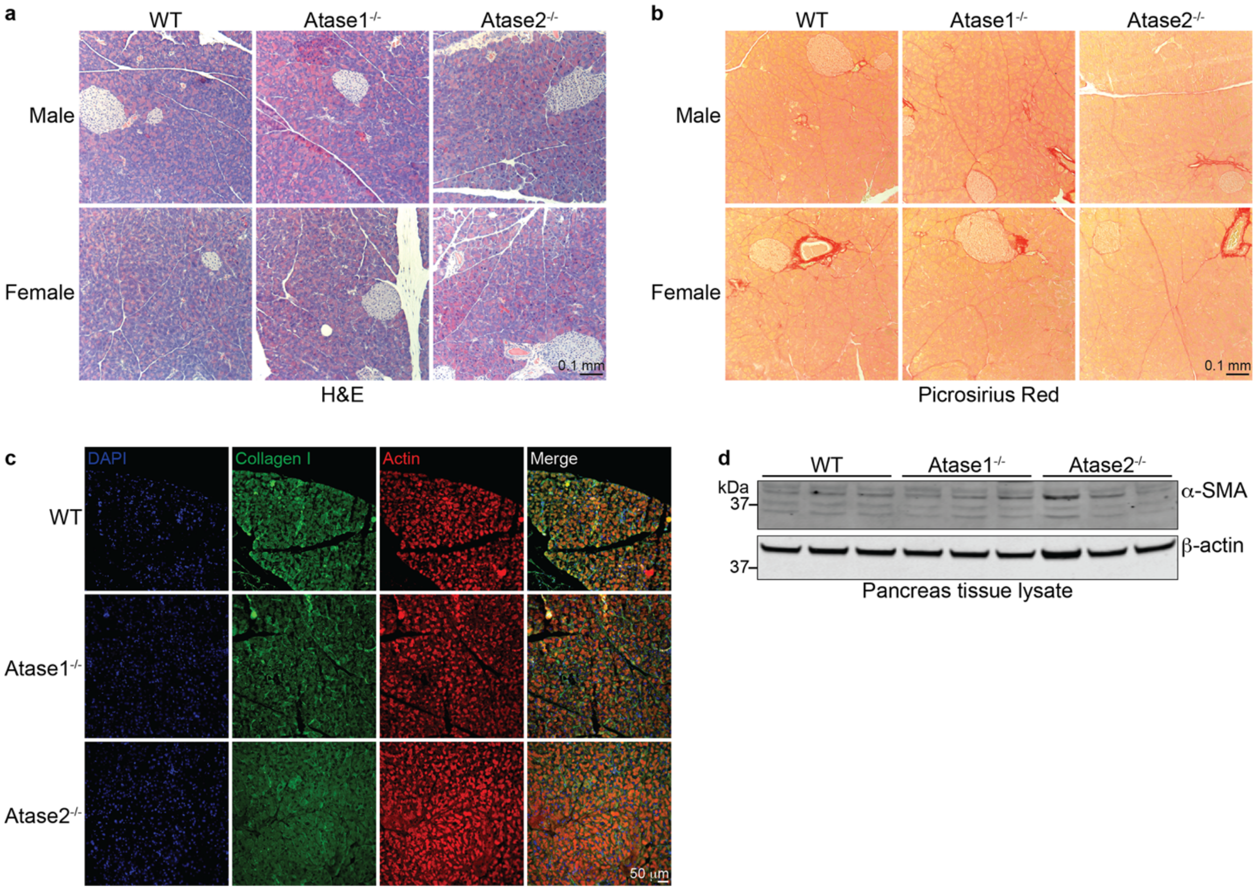

Supplemental Figure 3. Atase1$^{-/-}$ and Atase2$^{-/-}$ mice do not have signs of pancreatic disease. (a) Bright-field images of hematoxylin and eosin staining of 5 µm paraffin-embedded tissue. (b) Bright-field images Picrosirius red staining of 5 µm paraffin-embedded tissue. (c) Immunofluorescence of male pancreatic sections stained for collagen I and actin (via phalloidin-TRITC). (d) Western blotting of pancreatic lysate for α-smooth muscle actin (SMA). Mice are 6-7 months of age at time of study.

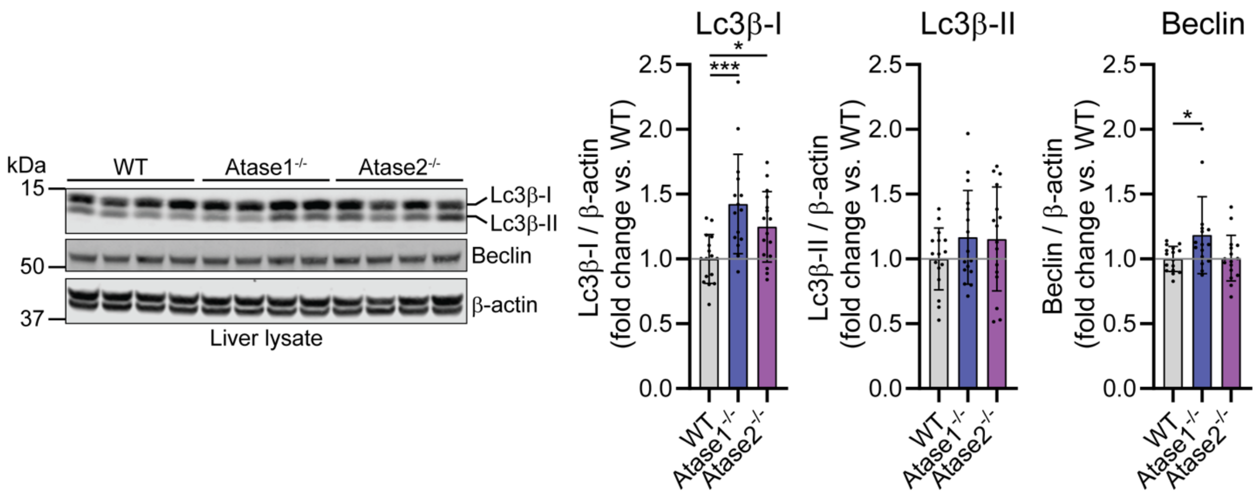

Supplemental Figure 4. Atase1$^{-/-}$ and Atase2$^{-/-}$ mice exhibit chronic activation of macroautophagy in liver. Western blotting of liver lysates for autophagy markers Lc3β and Beclin. Representative images are shown. Data are mean ± SD, n = 16 animals per genotype. *p < 0.05, ***p < 0.0005 via ordinary one-way ANOVA with Dunnett's multiple comparison test.

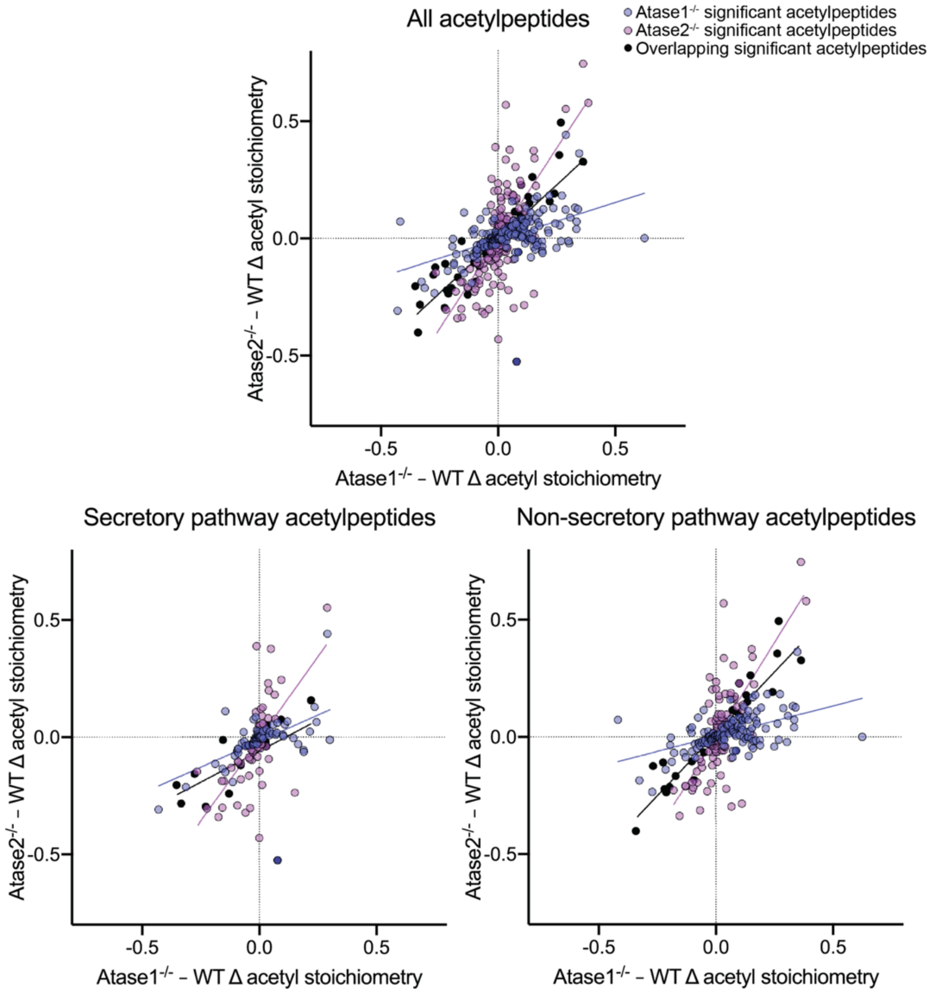

Supplemental Figure 5. Atase1$^{-/-}$ and Atase2$^{-/-}$ stoichiometry of acetylation reveals significant overlap. Scatterplots of acetylpeptides that exhibited a statistically significant difference in acetyl stoichiometry from WT. The acetylpeptides with changes that were only statistically significant in Atase1$^{-/-}$ (blue), Atase2$^{-/-}$ (purple), and both (black) are shown with their corresponding simple linear regression line. *F*-test results under the null hypothesis that the overall slope is zero are as follows along with Pearson's correlation coefficient (*r*): all acetylpeptides: Atase1$^{-/-}$ $p < 0.0001$ (0.507), Atase2$^{-/-}$ $p < 0.0001$ (0.690), overlapping $p < 0.05$ (0.806); secretory pathway: Atase1$^{-/-}$ $p < 0.0001$ (0.501), Atase2$^{-/-}$ $p < 0.0001$ (0.643), overlapping $p < 0.05$ (0.496); non-secretory pathway: Atase1$^{-/-}$ $p < 0.0001$ (0.518), Atase2$^{-/-}$ $p <$ 0.0001 (0.704), overlapping $p < 0.05$ (0.925).

a

Gene	Protein name	Site	Detected in Atase1$^{-/-}$?	Atase1$^{-/-}$ acetyl stoichiometry (*n*)	Detected in Atase2$^{-/-}$?	Atase2$^{-/-}$ acetyl stoichiometry (*n*)
Rpl27	60S ribosomal protein L27	K107	Yes	0.7703 (3)	Yes	0.8502 (2)
Scp2	Non-specific lipid-transfer protein	K132	Yes	0.3992 (3)	Yes	0.4044 (1)
Ass1	Argininosuccinate synthase	K165	Yes	0.0993 (3)	Yes	0.1705 (2)
Rps16	40S ribosomal protein S16	K109	Yes	0.0113 (3)	Yes	0.0136 (4)
Txndc5	Thioredoxin domain-containing protein 5	K129	Yes	0.0063 (1)	Yes	0.0166 (3)
Prkcsh	Glucosidase 2 subunit beta	K167	Yes	0.0029 (1)	Yes	0.0038 (3)
Ces3a	Carboxylesterase 3A	K54	Yes	0.0026 (1)	Yes	0.0022 (3)
Atl2	Atlastin-2	K314	Yes	0.0020 (2)	Yes	0.0065 (3)
Uba52	Ubiquitin-60S ribosomal protein L40	K48	Yes	0.0003 (1)	Yes	0.0020 (3)
Sec23ip	SEC23-interacting protein	K344	No	-	Yes	0.1383 (4)
Rps6	40S ribosomal protein S6	K143	No	-	Yes	0.0008 (3)

b

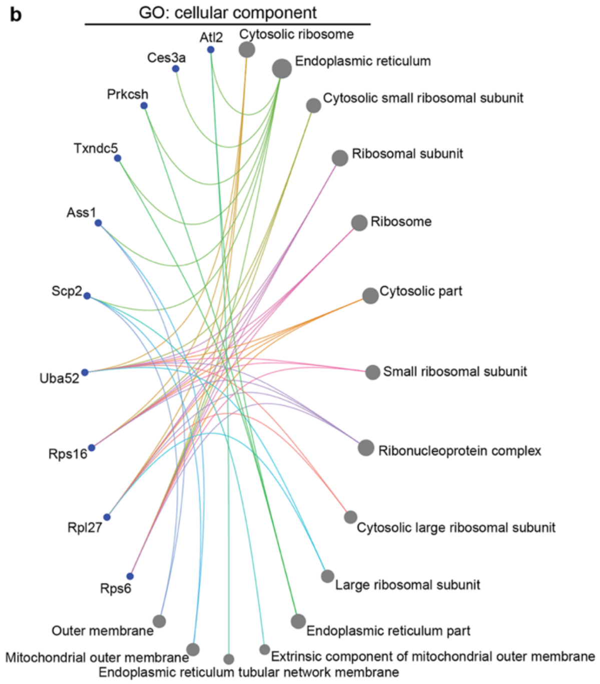

Supplemental Figure 6. Atase1$^{-/-}$ and Atase2$^{-/-}$ create new acetylation sites in multiple secretory proteins. (a) Acetylation sites that are detected and quantified in either Atase1$^{-/-}$ or Atase2$^{-/-}$ liver ER ($n \geq 3$ mice) but undetected in WT liver ER. Only acetylpeptides that have an identified subcellular localization of "ER," Golgi," or "secreted" are included. (b) Gene ontology (GO) cellular component overrepresentation analysis of the deacetylated proteins shown in panel a. The dot size of each network category is scaled by number of proteins included in that category. The top 15 enriched categories are shown that have been filtered by a false discovery rate threshold of $q = 0.05$.

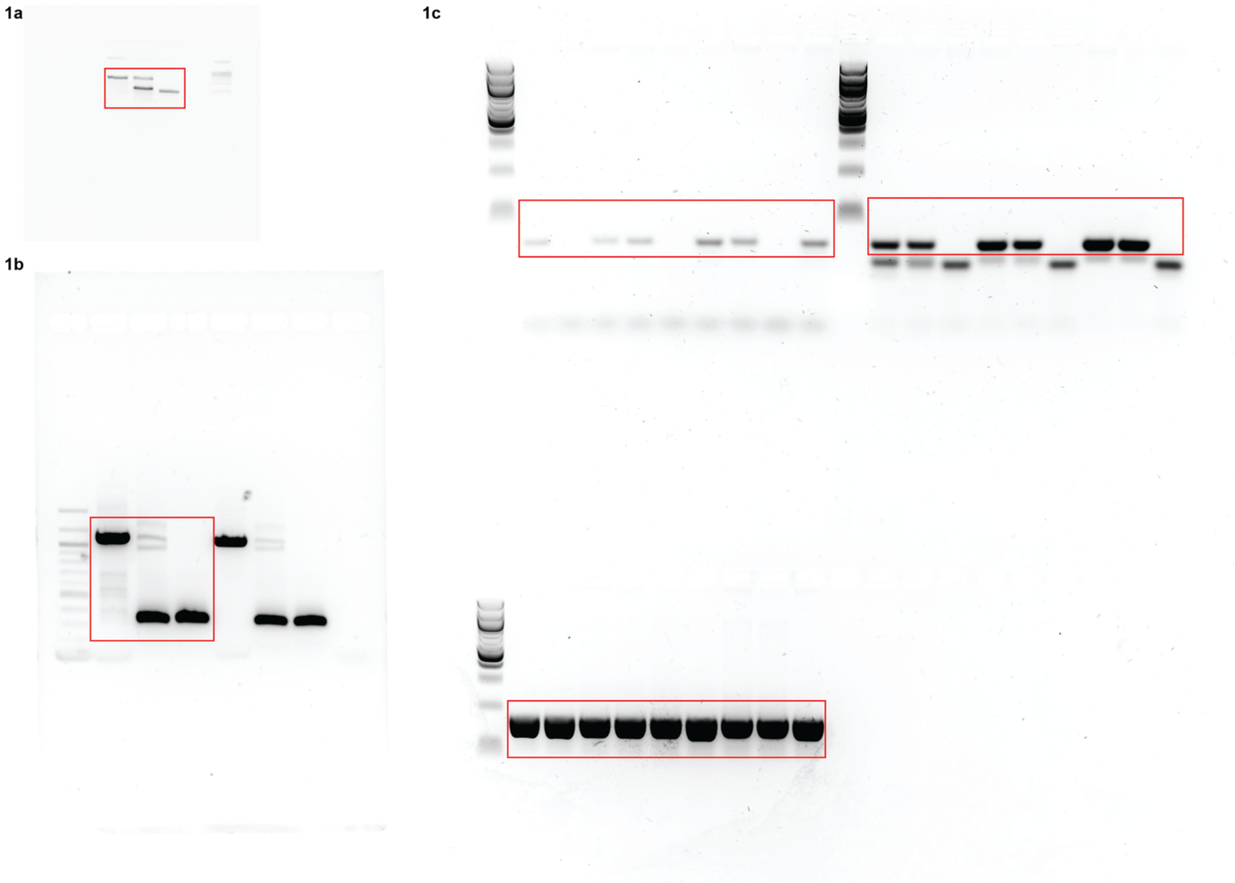

Supplemental Figure 7. Uncropped Gels from Figure 1.

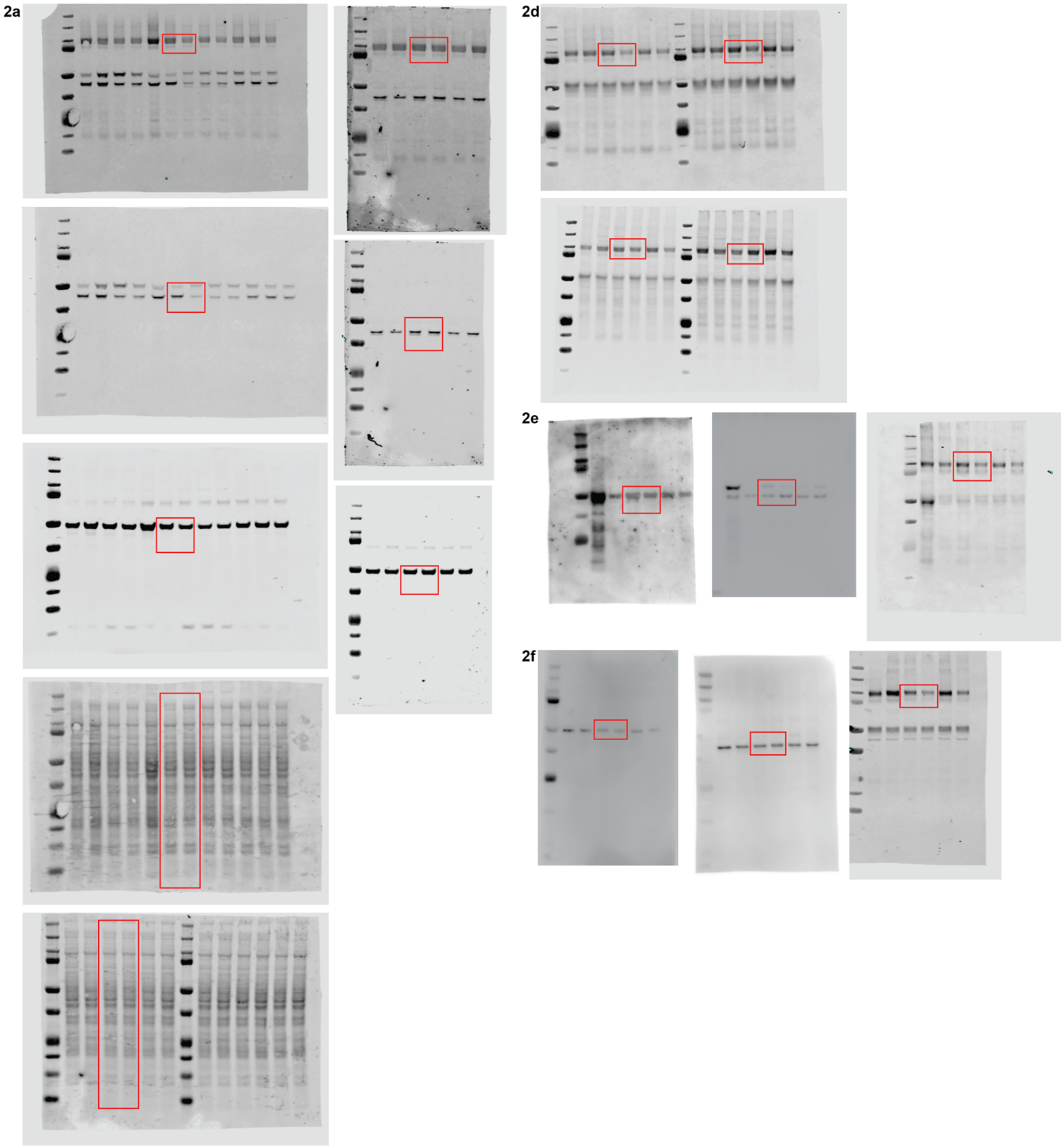

Supplemental Figure 8. Uncropped Western Blots from Figure 2.

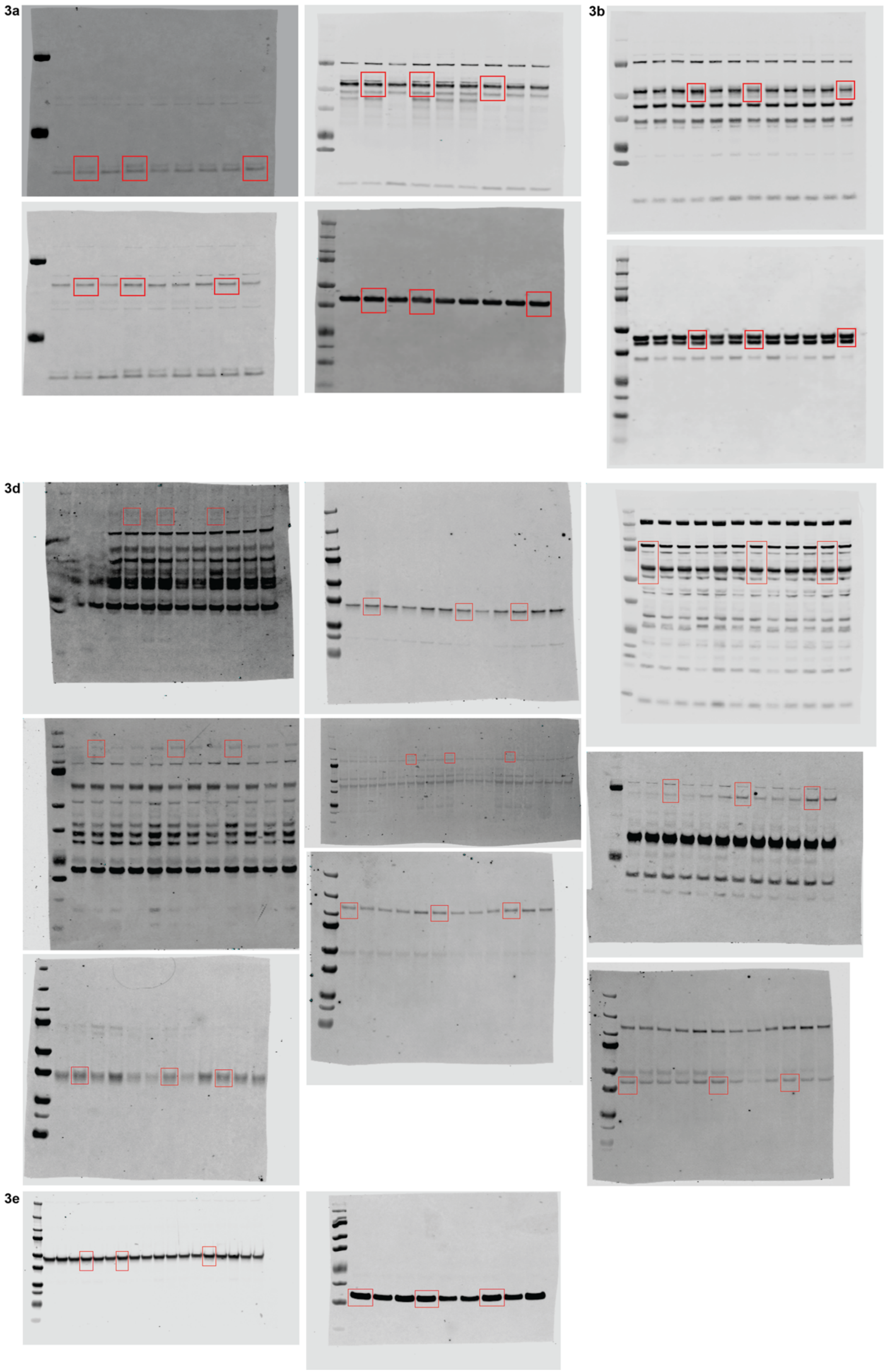

Supplemental Figure 9. Uncropped Western Blots from Figure 3.

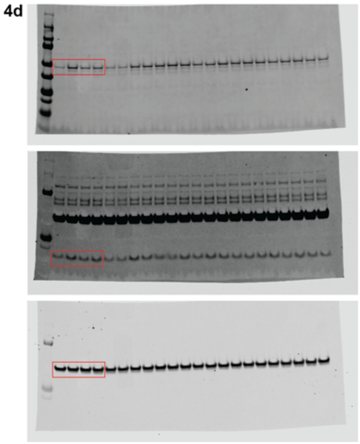

Supplemental Figure 10. Uncropped Western Blots from Figure 4.

S3d

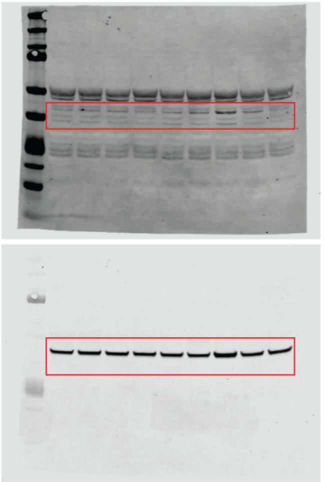

Supplemental Figure 11. Uncropped Western Blots from Figure S3.

S4

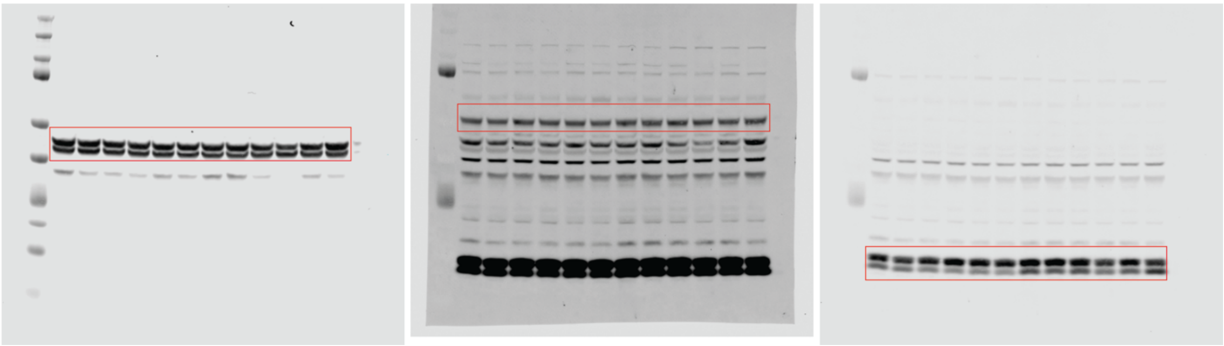

Supplemental Figure 12. Uncropped Western Blots from Figure S4.
