# Supplementary material for: Endoplasmic reticulum acetyltransferases Atase1 and Atase2 differentially regulate reticulophagy, macroautophagy and cellular acetyl-CoA metabolism
Source: Commun Biol. 2021 Apr 12;4:454. doi: 10.1038/s42003-021-01992-8 (PMC8041774; doi:10.1038/s42003-021-01992-8)
Supplement: Supplementary file 3 — Description of Additional Supplementary Files [file 42003_2021_1992_MOESM3_ESM.pdf]

Description of Additional Supplementary Files

File name: Supplementary Data 1

Description: Source data underlying graphs shown in figures and supplementary figures.
